# The role of organizational culture in nurse wellbeing and work-related psychosocial outcomes: a systematic review

**DOI:** 10.1186/s12912-026-05086-w

**Published:** 2026-07-22

**Authors:** Farzaneh Amini, Seyedeh Somayeh Kazemi

**Affiliations:** 1 Department of Biostatistics and Epidemiology, Student Research Committee, Faculty of Health, Mazandaran University of Medical Sciences, Sari, Iran; 2 Department of Health Education & Health Promotion, Faculty of Health, Mazandaran University of Medical Sciences, Sari, Iran; 3 Orthopedic Research Center, Sari Imam Khomeini Hospital, Mazandaran University of Medical Sciences, Sari, Iran

**Keywords:** Organizational culture, Nurse wellbeing, Job satisfaction, Burnout, Psychosocial outcomes, Systematic review

## Abstract

**Background:**

Nurses are a cornerstone of global healthcare systems, yet nursing organizations face persistent challenges including workforce shortages and burnout. With the World Health Organization projecting a global deficit of 4.5 million nurses by 2030, fostering a positive organizational culture and enhancing professional wellbeing are critical strategies for nurse retention. This systematic review aims to synthesize evidence on the impact of organizational culture on nurse wellbeing and related psychosocial outcomes (including happiness, job satisfaction, burnout, and psychological health), identify key moderating factors, and propose evidence-based interventions.

**Methods:**

Following a protocol registered in PROSPERO (CRD420261377119), we conducted a systematic review in accordance with the Cochrane Handbook and PRISMA guidelines. A comprehensive search was performed in January 2026 across PubMed, Scopus, and Web of Science using the PEO framework. After screening, 29 studies were included.

**Results:**

A thematic synthesis of 29 studies indicated that a positive organizational culture characterized by trust, social support, and justice was generally associated with higher nurse happiness and lower burnout. However, it is important to note that the included studies were predominantly cross-sectional with low to moderate methodological quality. There was significant heterogeneity in the measurement tools used for organizational culture and wellbeing outcomes, which limits the ability to pool results quantitatively. Contextual stressors such as heavy workloads were consistently identified as attenuating factors.

**Conclusion:**

While the current evidence base is of low quality and characterized by high heterogeneity, the synthesized findings suggest that fostering a positive, ethical, and supportive organizational culture is likely a key factor in enhancing nurse happiness and retention. Due to the limitations of the existing literature (primarily cross sectional designs), these associations should not be interpreted as causal. Healthcare leaders should consider cultural interventions as part of a broader strategy to improve workforce wellbeing, while prioritizing future longitudinal research to establish robust evidence for policy making.

**Supplementary Information:**

The online version contains supplementary material available at https://doi.org/10.1186/s12912-026-05086-w.

## Introduction

Nurses, as the cornerstone and an essential human resource within healthcare systems, play a pivotal role in shaping the global health landscape. Consequently, prioritizing their health and wellbeing is fundamental to safeguarding worldwide public health [1, 2]. Nurses are not only instrumental in enhancing health outcomes and strengthening the health economy, but investment in this workforce is also imperative for achieving sustainable healthcare systems [3]. Nonetheless, nursing organizations and managers persistently grapple with challenges such as workforce shortages and burnout [4]. World Health Organization projections indicate a global shortfall of 4.5 million nurses by 2030 [5, 6]. Furthermore, the International Council of Nurses estimates a global need for approximately 10.6 million additional nurses to address the deficit, a gap exacerbated by an aging nursing population and existing workforce shortages [7]. Therefore, it is critical for governments and healthcare organizations to implement necessary measures to reduce turnover rates and ensure nurse retention. To effectively retain nurses, the focus must be on enacting longterm strategies that safeguard their safety and wellbeing and bolster the nursing workforce. These challenges underscore the urgent need to focus on nurse retention strategies, including the enhancement of organizational culture and the promotion of professional wellbeing [8].

In the pursuit of safeguarding nurse health and advancing high quality nursing care for the global population, wellbeing has been identified as a critical factor in the work environment, exerting a positive influence on nurses’ health and welfare [1]. According to Seligman’s (2011) model, workplace happiness is defined as a composite of positive emotions, a sense of meaning in one’s work, and positive relationships [9]. This construct is fundamentally distinct from job satisfaction, which primarily pertains to a cognitive evaluation of working conditions [10]. Workplace enjoyment and happiness can be characterized as predictive factors for a workforce, enabling purpose and meaning to positively impact healthcare professionals’ work [11, 12] and potentially serving as an antidote to burnout and stress [13]. Studies indicate that nurses’ wellbeing directly affects not only their psychological health but also the quality of patient care and the reduction of medical errors [12, 14]. For instance, research by Diener and Seligman (2017) emphasizes that supportive work environments can enhance nurses’ wellbeing by fostering a sense of belonging and meaningful work [10].

Organizational culture is a pivotal component in shaping the work environment and influencing employees’ behavior, emotions, and wellbeing, holding particular significance within the healthcare sector [15, 16]. It comprises a system of shared meanings, encompassing social relationships with both management and colleagues in terms of trust and perceived appreciation [17, 18]. This system of shared meaning is defined by the underlying values, beliefs, and behavioral norms that guide employee conduct [19]. A growing body of research indicates that a positive organizational culture can significantly enhance nurse happiness. For instance, Schein (2010) posits that organizational culture constituted by the shared values, beliefs, and behaviors within an organization directly influences how staff interact with one another and with patients [20]. When this culture is founded on mutual support, trust, and respect, it can substantially contribute to nurses’ professional wellbeing and job satisfaction [21]. Numerous studies demonstrate that a positive organizational culture fosters nurse wellbeing by enhancing trust, social support, and job flexibility [18, 20]. Illustratively, the research by Bakker & Demerouti (2017) found that robust social support and effective communication in the workplace can mitigate stress and burnout. Their study showed that antistress training programs and open managerial dialogues led to a 30% reduction in nurse burnout rates. Nurses operating in environments with a positive culture typically report a greater sense of belonging and value, which in turn enhances their overall wellbeing [22]. Furthermore, organizational culture profoundly impacts nurse motivation and commitment. Evidence suggests that nurses working in environments with a strong, positive culture exhibit higher retention rates and lower incidences of burnout [23]. This, consequently, contributes to improved quality of healthcare services and increased patient satisfaction [24].

Nevertheless, several challenges can modulate the influence of organizational culture on nurse wellbeing. A primary challenge is high work pressure and excessive task loads, which can precipitate burnout and diminish wellbeing. This underscores the critical need to balance workload demands with available organizational resources. Furthermore, the persistence of negative organizational cultures, characterized by a lack of collegial support or poor communication, can significantly undermine nurses’ happiness [22]. Despite these challenges, significant opportunities exist to enhance organizational culture and, consequently, nurse wellbeing. For instance, implementing professional development and training programs can empower nurses by strengthening their competencies, thereby fostering a greater sense of professional fulfillment and job satisfaction. Similarly, fostering robust internal communication and cultivating an environment of support and trust can effectively transform the organizational culture, leading to a marked improvement in nurse happiness [20].

Given the World Health Organization’s projections of a severe impending global nursing shortage, fostering a positive and supportive organizational culture is an essential strategy for nurse retention and recruitment. This systematic review aims to synthesize evidence on the impact of organizational culture on nurse wellbeing and related psychosocial outcomes (including happiness, job satisfaction, burnout, and psychological health), identify key moderating factors, and propose evidence-based interventions. The findings of this study will provide policymakers with a foundation for prioritizing targeted initiatives, such as designing agile work environments and implementing stress management training. Through such measures, it will be possible to enhance working conditions and elevate nurse wellbeing, ultimately leading to improved clinical outcomes, greater patient satisfaction, and an elevated standard of healthcare quality.

## Method

### Review questions

- What is the impact of organizational culture on nurse wellbeing and related psychosocial outcomes (including job satisfaction, burnout, and psychological health) among nurses?
- Based on the existing evidence, what recommendations focusing on organizational culture can be derived to transform nursing work environments and promote nurse wellbeing and happiness?

### Study design

This systematic review is a part of a project with the ethics code IR.MAZUMS.IMAMHOSPITAL.REC.1402.119. Before it was conducted, a priori protocol for the research was registered in PROSPERO with registration identification number (CRD420261377119) in 23 April 2026. The methodology was guided by the JBI Manual for Evidence Synthesis [25] and reported in accordance with the PRISMA 2020 statement [26].

### Search strategy and selection criteria

To identify published articles for inclusion in this study, a comprehensive literature search was conducted in January 2026 in the following databases: PubMed, Scopus, Web of Science. The complete search strategies for all databases, including Boolean operators, subject headings, keywords, limits, language restrictions, and the exact search date, are provided in supplementary file. The databases PubMed, Scopus, and Web of Science were selected for their comprehensive coverage of nursing and healthcare literature, and their widespread use in systematic reviews in this field. The research question followed the PEO framework [27] which stands for (P)population (nurses), (E)exposure (organizational culture), and (O)outcome (happiness/wellbeing). The search method employed the Boolean operator OR between key synonym words, which were then linked with AND to complete the search as shown in the supplementary material (Table 1).

**Table 1:** Description of the PEO framework elements

Description of the PEO (*P* = Population, E = Exposure, O = Outcome) elements	
Population (P)	Nurses with standard working experience in healthcare settings (e.g., hospital nurses or specialized units).
Exposure (E)	Organizational culture constructs (e.g., transformational leadership, perceived support, justice).
Outcome (O)	Nurse happiness, wellbeing, job satisfaction, burnout, or related psychosocial outcomes

### Inclusion and exclusion criteria

All studies had to meet the following criteria to be included: be from any country and conducted on nursing personnel of any cadre as the study population, be original articles in English, used any study design, no limitation to the year of publication, nurse happiness/wellbeing was the outcome of the study, and the outcome was related to organizational culture or its variables. We excluded conference papers, other systematic literature reviews, metaanalyses, letters of opinion, editorials, essays, case studies, and literary works. Additionally, we did not consider comments or narrative articles, as well as full text articles written in languages other than English. Furthermore, studies without nurse participants or those involving a mixed group of nurses and other professionals where nurse specific results could not be extracted were also excluded.

### Screening of the articles

The database search results were imported into EndNote to compile all records and subsequently exported into the Cochrane software for deduplication and screening. Two reviewers (SSK and FA) independently and blindly screened titles and abstracts. Disagreements were resolved through discussion. Then, full text screening was performed to evaluate each study for eligibility for inclusion. Disagreements regarding the articles were resolved through discussion and consensus during screening. In situations where no agreement was reached, a third screener was involved.

### Data extraction and data synthesis

The following data from the included studies was extracted into a standardized Excel sheet for analysis: the primary author, year of publication, the country in which the research was conducted, the main study objective/ aim, study design, sample size, study population, study setting, response rate, exposure details (organizational culture type or variable), the measurement instruments employed, outcome details (nurse happiness/wellbeing), the measurement instrument used, and the significant findings. To ensure accuracy and minimize bias, data extraction was performed independently by two reviewers (SSK and FA). Any discrepancies in the extracted data were resolved through discussion between the two reviewers; if consensus could not be reached, a third reviewer was consulted. This independent dual extraction process was applied to all included studies. Tables were created to organize the extracted information. Data were synthesized using thematic synthesis following the approach of Thomas & Harden (2008) [28] inductive line-by-line coding of study findings, development of descriptive themes, and generation of analytical themes across studies. Heterogeneity in constructs and measurement instruments was handled by grouping findings at the level of the underlying construct rather than the specific instrument, and study quality was considered when weighing the strength of thematic claims. Meta-analysis was not undertaken owing to substantial clinical and methodological heterogeneity across studies, including divergent operationalization of both organizational culture and happiness/wellbeing.

### Quality assessment of included studies

The methodological quality of the included studies was assessed using standardized critical appraisal tools from the Joanna Briggs Institute (JBI) [29]. Cross sectional studies were appraised using the JBI Checklist for Analytical Cross Sectional Studies [30], qualitative studies using the JBI Checklist for Qualitative Research [31]. Theoretical and commentary papers were synthesized narratively without a formal quality appraisal. Two reviewers independently assessed each study. Any discrepancies in the quality ratings were resolved through discussion or by consulting a third reviewer.

## Results

### Search results and study selection

**Fig. 1:**
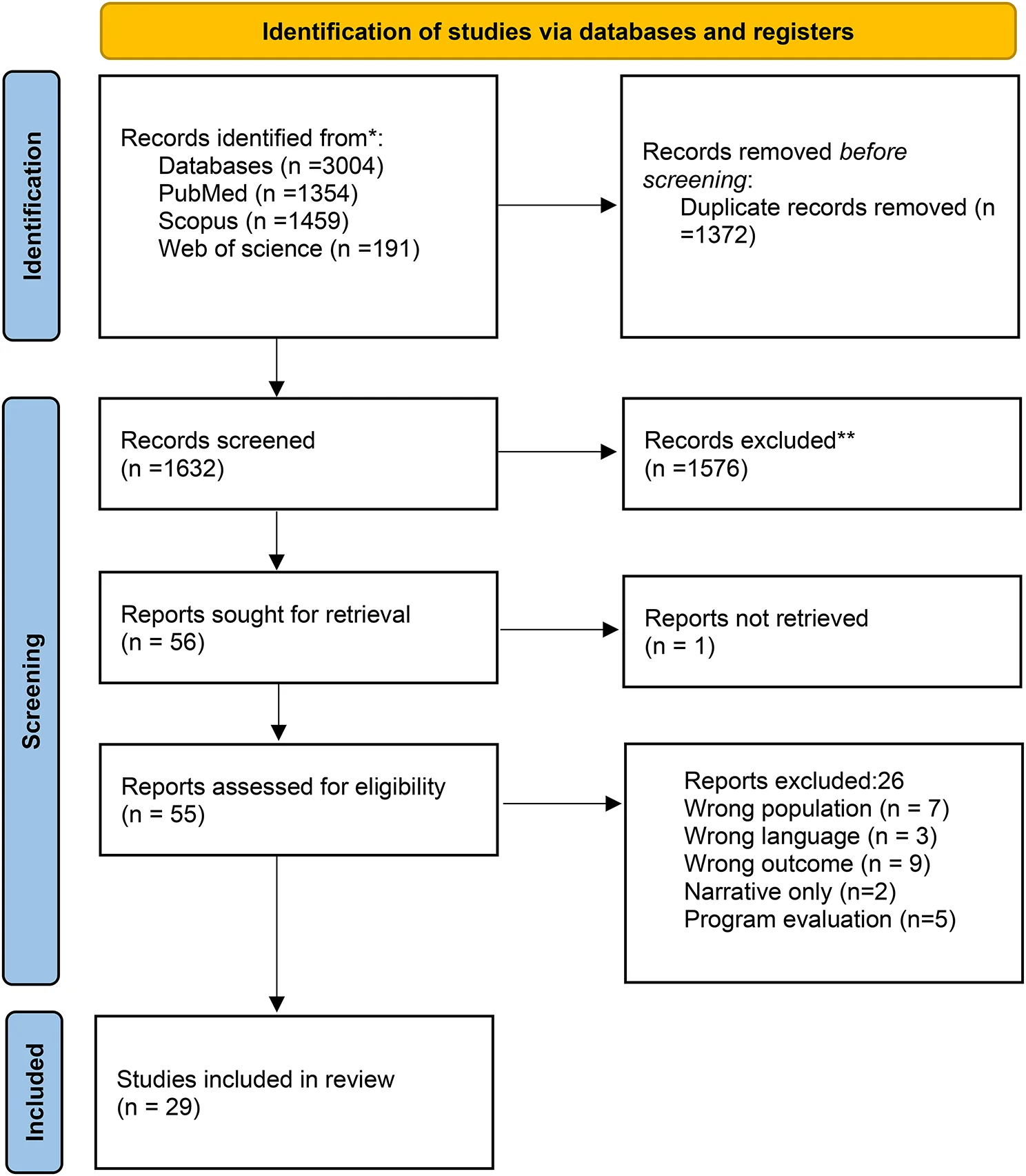
PRISMA flow diagram of the selected studies

The systematic search and selection process are detailed in the PRISMA flow diagram (Fig. 1). A total of 3004 records were identified from the searched databases: PubMed (*n* = 1354), Scopus (*n* = 1459), and Web of Science (*n* = 191). After removing 1372 duplicate records, the titles and abstracts of 1632 unique articles were screened for eligibility. Following this initial screening, 55 fulltext articles were assessed. Of these, 26 articles were excluded for the following reasons: wrong population (*n* = 7), wrong outcome (*n* = 9), program evaluation (*n* = 5), narrative only (*n* = 2), and wrong language (*n* = 3). Consequently, 29 studies met the inclusion criteria and were included in the final qualitative synthesis. No additional records were identified through other sources.

### Characteristics of included studies

The detailed characteristics of the 29 included studies are presented in Table 2. The studies encompassed a diverse geographical spread, including Egypt, Turkey, China, the United States, Canada, Australia, Brazil, South Korea, Italy, the Netherlands, Taiwan, Sweden, Romania, Iran, Finland, and the United Kingdom. The vast majority of the studies (*n* = 26) utilized a crosssectional design, while three studies employed a qualitative methodology. Sample sizes varied considerably, ranging from 52 to 3,156 nurse participants, with the settings primarily being hospitals of various types (e.g., public, university, Magnet recognized, long term care). The organizational culture constructs examined were multifaceted and measured using various validated instruments, such as the Perceived Organizational Support (POS) Scale, Global Transformational Leadership Scale, Organizational Justice Scale, Innovation Culture Scale, and the Person Organization Fit Scale, among others. Similarly, nurse happiness/wellbeing was assessed using a range of outcome measures, including the Workplace Happiness Scale, Oxford Happiness Questionnaire, Job Satisfaction scales, Vitality scale, and measures of burnout and psychological wellbeing.

### Synthesis of findings

The narrative synthesis of findings from the 29 studies consistently demonstrated a significant relationship between organizational culture and nurse happiness. Key themes emerged, highlighting that a positive organizational culture is strongly associated with enhanced nurse wellbeing. Constructs such as perceived organizational support, transformational leadership, organizational justice, a positive team climate, and a good person organization fit were repeatedly identified as positive contributors to nurse happiness, job satisfaction, and work engagement. For instance, studies by Ibrahim et al. (2023) and Laschinger et al. (2011) found transformational leadership and structural empowerment to be significant positive predictors of job satisfaction and lower burnout. Conversely, elements of a negative organizational culture, such as workplace bullying, inadequate support, and a punitive climate, were correlated with higher levels of stress, burnout, absenteeism, and turnover intentions, as evidenced in studies by Brewer et al. (2020) and Carter et al. (2013). Furthermore, several studies identified mediating variables; for example, Li et al. (2024) reported that work wellbeing partially mediated the relationship between perceived organizational support and medical narrative ability, and Karadas et al. (2025) found workplace happiness mediated the link between person organization fit and job embeddedness. The qualitative studies by Gould et al. (2024) and Geanellos (2005) enriched these findings by emphasizing the profound impact of a supportive, respectful, and positive work environment on nurses’ mental health and overall sense of wellbeing.

**Table 2:** Characteristics of the included studies (*n* = 29)

NO	Author/ year& country	Study Design	Population / Sample Size / Response Rate	Setting	Exposure Measure(s)	Main outcome	Outcome Measure(s)	Key Findings
1	Abdelmotaleb & Saha (2020), Egypt [32]	Crosssectional (Twowave survey)	Nurses (*n* = 349) / Overall response rate: 59%	Large public hospital, Egypt	SRHRM scale (Shen & Benson, 2016)	Employee Vitality (Eudaimonic wellbeing) & Positive Affect (Hedonic wellbeing)	Vitality scale (Kark & Carmeli, 2009); Positive Affect scale (Warr, 1990)	SRHRM positively influences perceived organizational morality, which in turn increases positive affect at work, ultimately leading to higher employee vitality. A sequential mediation model was supported.
2	Gould et al. (2024), United Kingdom [33]	Qualitative Thematic Analysis	Healthcare Workers (HCWs) (*n* = 60 text conversations) / Not applicable (Purposive sampling from a larger dataset)	UKwide (NHS) via textbased support service	Not applicable (Qualitative analysis of concerns)	Mental Health & Wellbeing during COVID19 pandemic	Thematic analysis of anonymised text conversations	Three primary themes emerged: (1) Poor mental health (overwhelming negative feelings, active crisis), (2) Negative work experiences (negative culture, inadequate support, trauma, abuse), (3) Impact of COVID19 (additional work pressure, isolation & risk). Highlights severe distress, organizational stressors, and the need for continued mental health support.
3	O’Hara et al. (2025), USA [34]	Crosssectional Descriptive Correlational	Nurses, Physicians, Allied Health Professionals (*n* = 199) / Response rate: 11.2% (from 331 initiations)	A Magnetrecognized health system in the Northeastern United States	Innovation Culture Scale (10item), Perceived Wellness Culture and Environment Support Scale (PWCESS), Organizational Culture and Readiness Scale for EBP (OCRSIEP) Short Version	Clinician Wellbeing (Burnout, Mental Health, Job Satisfaction) & Healthy Lifestyle Behaviors	Job Satisfaction Scale (Price & Mueller), Perceived Stress Scale4 (PSS4), GAD2, PHQ2, SingleItem Burnout Scale, SelfRated Mental/Physical Health, Healthy Lifestyle Behavior Questions	Strong, significant correlations found among innovation, wellness, and EBP cultures. Higher ratings of each culture were significantly associated with higher job satisfaction, better selfrated mental/physical health, and lower stress, anxiety, depression, and burnout.
4	Li et al. (2024), China [35]	Crosssectional multicenter study	Nurses (*n* = 1831) / Not explicitly reported	8 hospitals in China	Perceived Organizational Support Scale (POS)	Medical Narrative Ability	Medical Narrative Ability Scale (MNA)	Perceived organizational support positively correlates with medical narrative ability. Work wellbeing partially mediates this relationship (52.36% of total effect).
5	Zhao et al. (2024), China [36]	Crosssectional	Primary care Nurses (*n* = 175) from 38 Community Health Centres (CHCs)	CHCs in Shanghai, Shenzhen, Tianjin, and Jinan, China	Learning Orientation Scale (Organizational)	Selfdirected learning, Quality of care, Organizational commitment, Work stress	Selfreport questionnaires for each outcome (e.g., Selfdirected Learning Scale, Organizational Commitment Scale)	Organizational learning was positively and significantly associated with nurses’ selfdirected learning and selfreported quality of care. No significant association was found with organizational commitment or work stress.
6	Yeşildağ et al. (2025), Turkey [37]	Crosssectional	Healthcare professionals (*n* = 400)	5 hospitals in Trabzon, Turkey	ViolencePrevention Climate Scale	Workplace Happiness	Workplace Happiness Scale	A statistically significant positive relationship was found between the violenceprevention climate and workplace happiness (*r* = 0.392). Those who experienced violence and did not feel safe in the workplace had lower levels of happiness.
7	Karadas et al. (2025), Turkey [38]	Descriptive, Crosssectional	Nurses (*n* = 240) / Not explicitly reported (Monte Carlo power analysis indicated 100% power)	A public hospital in Balikesir province, Turkey	PersonOrganization Fit Scale (Netemeyer et al., 1997; Turkish adaptation by Turunc & Celik, 2012)	Job Embeddedness & Workplace Happiness	Global Job Embeddedness Scale (Crossley et al., 2007; Turkish adaptation by Akgunduz et al., 2016); Happiness at Work Scale (SalasVallina & Alegre, 2018; Turkish adaptation by Bilginoglu & Yozgat, 2020)	Personorganization fit significantly and positively affected workplace happiness (β = 0.5993) and job embeddedness (β = 0.2105 direct, β = 0.5955 total). Workplace happiness mediated the relationship between personorganization fit and job embeddedness (indirect effect β = 0.3850). Staff nurses, those with higher education, and those working overtime had lower levels of fit, happiness, and embeddedness.
8	Ibrahim et al. (2023), Egypt [39]	Crosssectional	Nurses (*n* = 527) / Response rate: 93.1% (from 566 initiations)	A general hospital under the Egyptian Ministry of Health	Global Transformational Leadership Scale (Carless et al., 2000); Organizational Justice Scale (Niehoff & Moorman, 1993)	Quality of Nursing Work Life	Quality of Nursing WorkLife Scale (Brooks, 2001)	Nurses perceived high transformational leadership but moderate organizational justice and QNWL. Transformational leadership (β = 0.350) and organizational justice (β = 0.451) were significant positive predictors of QNWL. Transformational leadership was also a strong predictor of organizational justice (β = 0.468).
9	Tomo & De Simone (2020), Italy [40]	Crosssectional	Patient care workers (*n* = 492) / Not explicitly reported	Three governmentfunded hospitals in Southern Italy	Slightly altered version of the Copenhagen Psychosocial Questionnaire (COPSOQ)	Employee Wellbeing (Individual, Workrelated, Organizational factors)	Normalized sum of factors from COPSOQ (Individual, Workrelated, Organizational aspects)	Physicians had the highest wellbeing (2.52), followed by nurses (2.43) and technicians (2.37). Different factors significantly affected each professional group. Headache and use of technology skills were common significant factors across all groups. Nurses’ wellbeing was significantly affected by need for autonomy, recognition, and sense of belonging.
10	den Boer et al. (2017), The Netherlands [41]	Crosssectional	Nurses (*n* = 153) / Response rate: 48%	11 healthcare organizations in the Southern Netherlands	Adapted PatientCentred Care Questionnaire	Nurses’ Wellbeing & Job Satisfaction	Social Production Function Instrument (SPFIL) for Wellbeing; 38item Job Satisfaction Questionnaire	Patientcentred care and cocreation of care were positively correlated with nurses’ wellbeing and job satisfaction. Both variables were predictors of wellbeing, and patientcentred care was a predictor of job satisfaction. The length of time in the present position was negatively related to job satisfaction and wellbeing.
11	Hwang (2019), South Korea [42]	Crosssectional	Nurses (*n* = 226) from 17 LTC hospitals	LongTerm Care (LTC) hospitals in five cities, South Korea	Organizational Culture Type Questionnaire (Han, 2001)	Nurses’ Happiness	Oxford Happiness Questionnaire (Hills & Argyle, 2002)	The happiness of LTC hospital nurses was not high. Higher happiness was associated with: a healthier estimated subjective health status, working in an organization with hierarchyoriented culture properties, higher job satisfaction regarding autonomy, and a lower number of task requests. Autonomybased job satisfaction exerted the greatest effect.
12	Brewer et al. (2020), USA [43]	Crosssectional (Online survey)	Nurses (*N* = 242) / Response rate not explicitly stated	Various nursing workplaces in the USA (Hospital & NonHospital)	Negative Acts QuestionnaireRevised for Nursing (NAQRUS)	Wellbeing (Burnout, Job Satisfaction, Absenteeism)	WellBeing Index (WBI), Job Satisfaction single item, Selfreported Absenteeism	Prevalence of weekly/daily bullying was 31%. Among nurses exposed to bullying, organizational betrayal increased odds of burnout, job dissatisfaction, and absenteeism. Organizational support decreased odds of job dissatisfaction and absenteeism.
13	Battistelli et al. (2016), Italy [44]	Crosssectional	Nurses (*n* = 352) / Hospital A: *n* = 160 (76.2%), Hospital B: *n* = 192 (83.5%)	Two urban hospitals in Italy (Central and Northern Italy)	Survey of Perceived Organizational Support (POS; 8item scale)	Selfcompetence	Selfcompetence Scale (6item scale)	Perceived Organizational Support (POS) was positively related to selfcompetence through affective commitment. Continuance and normative commitment did not mediate this relationship. POS was positively related to affective and normative commitment and negatively related to continuance commitment.
14	von Treuer et al. (2014), Australia [45]	Crosssectional	Nurses (*n* = 142) / 23% response rate (from 650 potential participants)	A metropolitan Melbourne hospital, Australia	Work Environment Scale (WESR; Moos, 1994) 10 subscales	Psychological health (Somatic, Anxiety, Social Dysfunction, Depression)	General Health Questionnaire (GHQ28)	Different organizational climates occurred across shifts. Permanent night shift workers had lower coworker cohesion and higher managerial control. Coworker cohesion and involvement predicted somatic problems. Work pressure predicted somatic complaints, anxiety, and total GHQ score. Shift work alone did not predict health outcomes; organizational climate factors accounted for the variation.
15	Rodwell & Munro (2013), Australia [46]	Crosssectional	Nurses and Midwives (*n* = 226) / 35.4% response rate (from 771 employees)	A private, notforprofit Australian maternity hospital	Job Control Scale (Karasek, 1985); Social Support Scale (Caplan et al., 1980); Organizational Justice Scale (Colquitt, 2001)	Wellbeing, Job Satisfaction, Organizational Commitment	GHQ12; Global Job Satisfaction Scale; Affective Organizational Commitment Scale (Allen & Meyer, 1990)	Supervisor support and organizational justice had significant relationships with wellbeing and job satisfaction. Job control predicted job satisfaction and commitment. Coworker support predicted wellbeing. Evidence for substitutability of resources (e.g., supervisor support, justice) in mitigating stress. Interaction effects found between job control & supervisor support and job demands & supervisor support on job satisfaction. Curvilinear relationships were observed for some predictors.
16	Rocha et al. (2014), Brazil [47]	Crosssectional	Nurses (*n* = 52), Nursing Technicians/Auxiliaries (*n* = 146) / Not explicitly stated for overall rate	A public hospital, São Paulo State, Brazil	Brazilian Instrument for Assessing Organizational Culture (IBACO)	Organizational Values and Practices (Cooperation, Hierarchy, Individualism, Wellbeing, Integration, Rewards, Relationships)	IBACO Factors and Subscales	Hierarchical rigidity, centralization of power, individualism, and competition were prevalent. Values related to worker wellbeing, satisfaction, and motivation were not highly valued. Organizational practices promoting interpersonal relationships, continuous education, and rewards were also undervalued. Traditional models of work organization sustained practices and determined the organizational culture.
17	Carter et al. (2013), United Kingdom [48]	Crosssectional (Questionnaire & Interviews)	NHS Staff (*n* = 2950) / ~46% response rate; Interviews (*n* = 43)	7 NHS Trusts, North East England, UK	Revised Negative Acts Questionnaire (NAQR); Selflabelled bullying definition	Prevalence of Bullying, Psychological Distress, Job Satisfaction, Intentions to Leave, Sickness Absence	NAQR; GHQ12; Job Satisfaction; Intentions to Leave; Selfreported Sickness Absence	20% of staff reported being bullied; 43% witnessed bullying. Bullying associated with lower psychological health, job satisfaction, and higher intentions to leave. Managers were the most common source. Barriers to reporting included fear of inaction, being labelled a troublemaker, and seniority of the bully. Workload pressures and organizational culture were contributing factors.
18	Bao et al. (2013), Spain [49]	Crosssectional correlational	Nurses (*n* = 234) / Response rate: 24.6%	One of the largest university hospitals in Spain	Triaxial model of values (Economicalpragmatic, Ethicalsocial, Emotionaldevelopmental)	Burnout, Selfrated health, Turnover intention, Accident propensity	ShiromMelamed Burnout Scale; Singleitem selfrated health; 3item turnover intention scale; Accident propensity questionnaire	Economical and Ethical value incongruence correlated with burnout. All three types of value incongruence correlated with turnover intention. Ethical and Emotional value incongruence correlated with accident propensity. Burnout mediated some relationships.
19	Carvalho et al. (2013), Brazil [50]	Descriptive, Crosssectional	Nurses (*n* = 52) / Not explicitly stated	A public hospital in the interior of the state of São Paulo	Brazilian Instrument for the Evaluation of Organizational Culture (IBACO)	Organizational Values and Practices (Cooperation, Hierarchy, Individualism, Wellbeing, Integration, Rewards, Relationships)	IBACO Factors and Subscales	Hierarchical rigidity, centralization of power, individualism, and competition were prevalent. Values related to worker wellbeing, satisfaction, and motivation were not highly valued.
20	Tsai (2011), Taiwan [51]	Crosssectional	Hospital Nurses (*n* = 200) / Response rate: 66.7% (from 300 distributed)	Two hospitals in Central Taiwan	Organizational Culture Scale (22 items); Leadership Behavior Scale (26 items)	Job Satisfaction	Job Satisfaction Scale (12 items)	Organizational culture was positively correlated with leadership behavior (β = 0.55) and job satisfaction (β = 0.66). Leadership behavior was positively correlated with job satisfaction (β = 0.33), even after controlling for organizational culture.
21	Laschinger et al. (2012), Canada [52]	Descriptive Correlational	New Graduate Nurses (< 3 years experience) in acute care (*n* = 420) / Response rate: 30%	Acute care hospitals in Ontario, Canada	Areas of Worklife Scale (Workload, Control); Negative Acts QuestionnaireRevised (Bullying); Practice Environment Scale; Psychological Capital Questionnaire	Burnout (Emotional Exhaustion), Work Engagement, Mental Health, Turnover Intentions	Maslach Burnout Scale (Emotional Exhaustion); Utrecht Work Engagement Scale; Mental Health Index5; Turnover Intentions Scale	The Job DemandsResources model was supported. Job demands (workload, bullying) predicted burnout and poor mental health. Job resources (supportive environment, control) predicted work engagement and lower turnover intentions. Psychological capital influenced both burnout () and engagement (+).
22	Laschinger et al. (2011), Canada [53]	Crosssectional, Multilevel	Staff Nurses (*n* = 3,156) from 217 hospital units / Response rate: 40%	217 units in 25 acute care hospitals, Ontario, Canada	LeaderMember ExchangeMultidimensional Model (LMXMDM); Conditions for Work Effectiveness QuestionnaireII (Structural Empowerment)	Burnout (Emotional Exhaustion, Cynicism), Job Satisfaction	Maslach Burnout InventoryGeneral Survey (Emotional Exhaustion, Cynicism subscales); 4item global Job Satisfaction measure	Unitlevel LMX quality positively influenced unitlevel structural empowerment (β = 0.25). Higher unitlevel LMX was associated with lower individual cynicism (β = 0.48) and higher job satisfaction (β = 0.55). Higher unitlevel structural empowerment was associated with lower individual emotional exhaustion (β = 0.17) and higher job satisfaction (β = 0.30). At the individual level, higher core selfevaluation was associated with lower emotional exhaustion and cynicism, which in turn predicted lower job satisfaction.
23	Dackert (2010), Sweden [54]	Crosssectional	Auxiliary nurses & nurses’ aides (*n* = 329) / 67%	Elderly care, Sweden	Team Climate Inventory (TCI)	Wellbeing & Stress	Warr’s Wellbeing Scales; General WellBeing Questionnaire (GWBQ)	Team climate for innovation positively correlated with wellbeing and negatively with stress. Wellbeing mediates between team climate and stress.
24	Rondeau & Wagar (2006), Canada [55]	Crosssectional (Mail Survey)	Directors of Nursing from Nursing Homes (*n* = 125 facilities) / Response rate: 43.3%	Longterm care organizations (Nursing Homes) in Western Canada	High Involvement Work Practices (HIWP) 10item additive scale Progressive DecisionMaking Culture (3item scale) Nursing Training (10item additive scale)	Magnet (EmployerofChoice) Strength Nurse Satisfaction Resident Satisfaction	Magnet Strength (7item scale) Nurse Satisfaction (3item scale) Resident Satisfaction (3item scale)	Magnet strength was significantly associated with higher nurse and resident satisfaction. Progressive decisionmaking culture and investment in nursing training were significant predictors of magnet strength, while the mere adoption of High Involvement Work Practices (HIWP) was not. Lower nursing vacancy rates predicted higher nurse and resident satisfaction.
25	Chakraborty & Ahire (2025), India [56]	Crosssectional Survey	Nurses from various departments (*n* = 108) / Not explicitly reported	A tertiary hospital in Pune, India	Work Environment Factors (assessed via the SWINGJ Scale)	Job Satisfaction WorkFamily Spillover (Negative & Positive)	SWINGJ Scale (WorkFamily Negative Spillover, FamilyWork Negative Spillover, WorkFamily Positive Spillover, FamilyWork Positive Spillover) Job Satisfaction Indicators (5point Likert scale)	Work environment factors significantly influenced job satisfaction. Job satisfaction levels varied by department (highest in Pediatric Ward, lowest in Pediatric ICU). Negative workfamily spillover was observed, but scores were lower than expected. Organizational support, workload management, and job demands were key influencers.
26	Claponea & Iorga (2023), Romania [57]	Crosssectional Study	497 Healthcare Specialists (367 medical staff, 130 nonmedical staff)	Public and private medical units in Romania	Maslach Burnout Inventory (MBIHSS), ECO System (Organizational Justice), Multidimensional Scale of Perceived Social Support (MSPSS), Jefferson Scale of Physician’s Lifelong Learning (JeffSPLL)	Burnout, Wellbeing, Organizational Justice, Social Support, Lifelong Learning	WHO Wellbeing Index (WHO5), MBIHSS subscales (Emotional Exhaustion, Depersonalization, Personal Accomplishment)	Medical staff had higher personal accomplishment, organizational justice, and wellbeing. Nonmedical staff had higher emotional exhaustion and depersonalization. Social support affects wellbeing through burnout dimensions, moderated by organizational justice. Lifelong learning was not a significant mediator.
27	Whitehead et al. (2023), Iran [58]	Crosssectional Study	600 Nurses from two specialized hospitals in Hamadan, Iran	Specialized hospitals in Hamadan, Iran	HealthPromoting Lifestyle Profile (HPLPII), Robbins’ Organizational Culture Questionnaire	Job Satisfaction, Quality of Life, Organizational Culture, Lifestyle	Minnesota Satisfaction Questionnaire (MSQ), 12Item Short Form Health Survey (SF12)	Lifestyle dimensions (spiritual health, physical activity, stress management, interpersonal communication), quality of life (physical, psychological), and organizational culture were significant predictors of job satisfaction. A structural equation model with high predictive power (GOF = 0.415) was validated.
28	Geanellos (2005), Australia [59]	Qualitative (Gadamerian hermeneutics)	12 clients (4 men, 8 women) / Not explicitly reported	Public and private hospitals in Greater Western Sydney, NSW, Australia	Not applicable (Qualitative exploration of nurse friendliness)	Client wellbeing and recovery	Thematic analysis of interviews	Nurse friendliness leads to mutuality, humour, fondness, reciprocity, and a lighthearted environment. It sustains clients, affirms them, and aids recovery.
29	ÅstedtKurki & Isola (2001), Finland [60]	Qualitative (Content analysis of diaries)	16 nurses (all female) / Not explicitly reported	Various healthcare settings in Finland (homes for older people, general hospitals, longstay wards)	Not applicable (Diaries of humour occurrences)	Use and impact of humour in nursing	Content analysis of diary entries	Humour between nurses and patients helps cope with unpleasant procedures and difficult situations. Humour among staff improves working climate and helps nurses manage work stress.

### Quality assessment of included studies

**Fig. 2:**
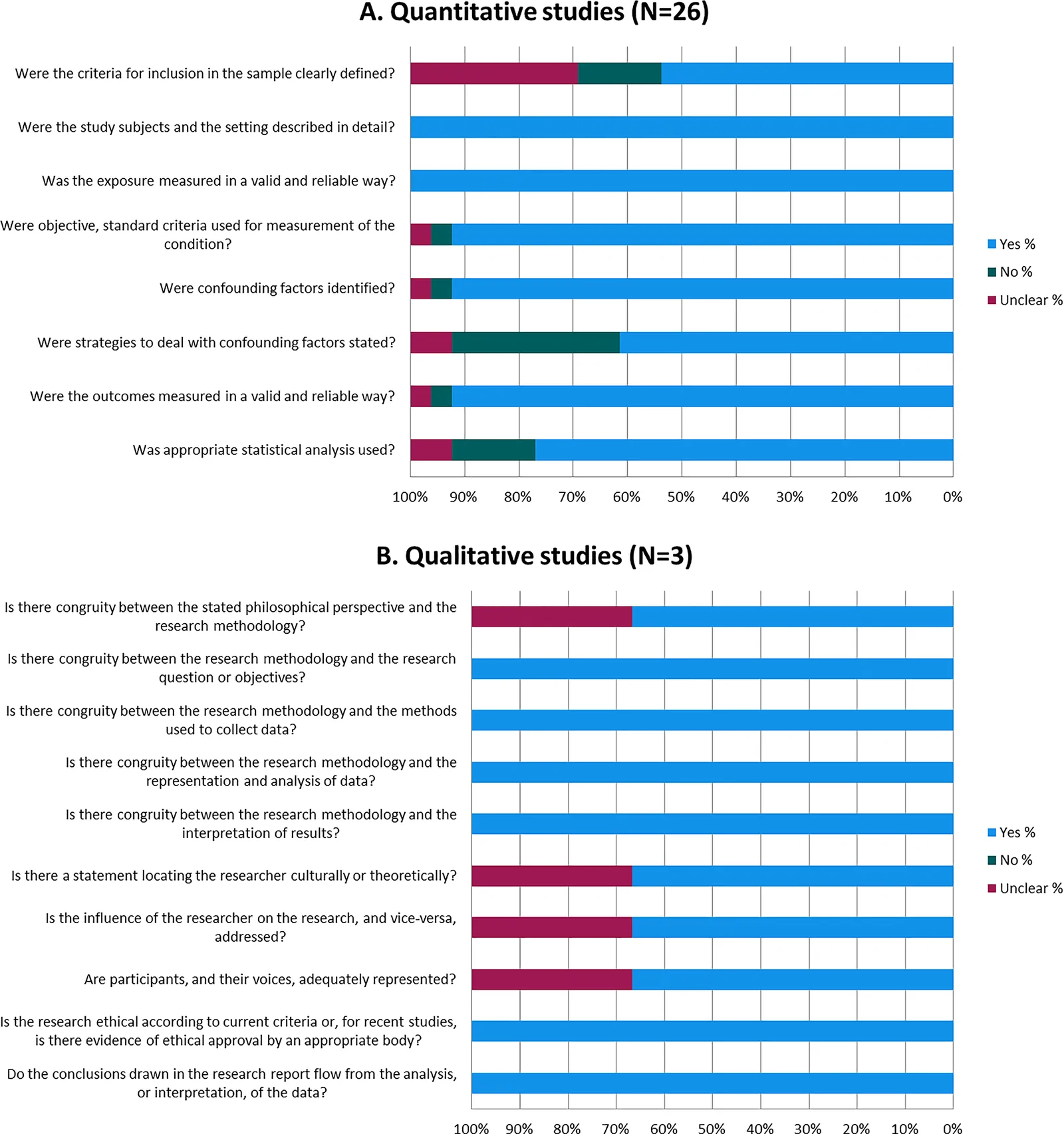
Quality assessment of the included studies

The methodological quality of the included studies was appraised using the Joanna Briggs Institute (JBI) critical appraisal tools, and the results are summarized in Fig. 2. For the cross sectional studies, most demonstrated clear criteria for inclusion, used valid and reliable measurement tools for both exposure and outcome, and employed appropriate statistical analyses. Common areas for potential bias or limitation included low or unreported response rates in some studies and the handling of confounding factors, which was not always explicitly addressed. The qualitative studies generally presented research questions and data collection methods clearly, with findings flowing logically from the data. The overall assessment indicated that the methodological quality of the included studies ranged from low to moderate. The vast majority of the evidence was derived from cross-sectional studies, which are inherently susceptible to recall bias, selection bias, and the inability to establish causality. Furthermore, response rates varied significantly across studies, and many failed to adequately control for potential confounders. Consequently, the overall certainty of the available evidence should be considered low because most included studies were cross-sectional and showed considerable methodological and clinical heterogeneity.

### Implications of low methodological quality and heterogeneity

It is crucial to acknowledge the low methodological quality of the included evidence and its implications for the conclusions drawn. The predominance of cross-sectional designs limits our ability to infer causal relationships between organizational culture and nurse happiness. The bidirectional nature of this relationship where happy nurses may also contribute to a positive culture cannot be disentangled from the current data. Furthermore, there was a high degree of clinical and methodological heterogeneity. Studies utilized diverse instruments to measure both organizational culture (e.g., OPQ, JSS, various leadership scales) and happiness/well-being (e.g., OHQ, SHS, burnout scales). This conceptual and measurement heterogeneity implies that the ‘positive culture’ and ‘happiness’ constructs were operationalized differently across contexts. The synthesis, therefore, represents a thematic aggregation of loosely related findings rather than a precise estimation of effect sizes. The low quality of evidence means that our recommendations are based on expert consensus and thematic trends rather than high certainty evidence. Therefore, while the direction of the association (positive culture → higher happiness) is consistent, the strength of this association is uncertain. Future research must prioritize longitudinal and experimental designs to strengthen the evidence base.

## Discussion

This systematic review synthesized evidence from 29 studies to explore the association between organizational culture and nurse wellbeing and related psychosocial outcomes. We included studies measuring a range of outcomes including happiness, job satisfaction, burnout, psychological health, and broader wellbeing indicators. While these constructs are conceptually distinct, they collectively reflect the psychosocial health of the nursing workforce and are frequently inter-related in the empirical literature. It is critical to note that the majority of included studies were cross-sectional, resulting in a body of evidence that is of low to moderate methodological quality. Consequently, while the thematic synthesis reveals consistent positive associations between supportive organizational cultures, nurse wellbeing, and nursing outcomes, these findings must be interpreted with caution. The observational nature of the data precludes definitive causal inferences, and the high degree of heterogeneity in study designs and measurement tools limits the precision of these estimates. The following discussion interprets these findings within the context of these methodological limitations.

### Methodological considerations in the synthesis

The thematic synthesis approach used in this review warrants several methodological considerations. First, the inductive coding process allowed themes to emerge directly from the data rather than being constrained by pre existing theoretical frameworks, which enhances the credibility of the findings. Second, the decision to group findings at the construct level rather than the instrument level was necessitated by the high degree of measurement heterogeneity observed across studies for example, organizational culture was measured using at least 12 different instruments across the 29 studies. While this approach enabled a coherent narrative synthesis, it also means that the themes represent broad conceptual categories rather than precise, instrument specific effects. Third, the weighting of thematic claims by study quality ensured that conclusions were not unduly influenced by low quality studies. However, given that the majority of included studies were cross sectional and of low to moderate quality, even the most robust themes are based on correlational evidence and should not be interpreted causally. Finally, the substantial clinical and methodological heterogeneity including differences in healthcare settings (acute care, long term care, primary care), geographical contexts, and nurse populations precluded meta-analysis and limits the generalizability of the findings. Nevertheless, the consistency of the thematic patterns across diverse contexts strengthens the plausibility of the observed associations and provides a foundation for hypothesis generation in future research.

### The centrality of a supportive and ethical culture

One of the more consistent findings across the included studies is the positive association between a supportive, ethical, and empowering organizational culture and favorable nurse outcomes. Key cultural facets such as transformational leadership [39, 53], organizational justice [39, 46, 57] and perceived organizational support [35, 44] emerged as frequently reported of nurse wellbeing and job satisfaction. These elements create an environment where nurses feel valued, treated fairly, and trusted, which may directly counteracts feelings of cynicism and emotional exhaustion [52]. This is further supported by studies on person organization fit [38] and a positive violence prevention climate [37], which show that when nurses’ personal values align with the organization’s and they feel physically and psychologically safe, their workplace happiness and embeddedness tend to significantly increase. These findings are considerably supported by the cross-sectional study conducted by Bilgin et al. (2025), which examined the relationship between attitudes toward brain drain and the work environment among 335 nurses working in university and city hospitals in Turkey. In that study, the mean score of nurses’ positive attitudes toward the work environment was considerably low (2.56 out of 4), whereas the mean attitude score toward brain drain was high (56.30 out of 80). The most notable finding of this study was that 35.7% of nurses who intended to work abroad cited unfavorable working conditions as their primary reason. This finding suggests that the absence of a supportive and ethical organizational culture may contribute to turnover intention and brain drain. However, given the cross-sectional design of the primary studies, we cannot rule out reverse causality, where unhappy nurses may also perceive the culture more negatively [61].

### The mediating role of resources and positive states

The relationship between culture and wellbeing is not merely direct; it is often suggested to be mediated by critical psychological and social resources. Our synthesis identifies workplace happiness [38] and work wellbeing [35] as potential mediators. For instance, a positive organizational culture is associated with higher levels of wellbeing, which in turn appears to be associated with improved professional outcomes such as like medical narrative ability. Furthermore, factors like social support, trust, and job control may act as buffers. Rodwell & Munro (2013) found that supervisor support and organizational justice could substitute for each other in mitigating stress, highlighting the potential importance of a resource rich environment. The findings of Dackert (2010) and O’Hara et al. (2025) reinforce that a culture promoting innovation, teamwork, and evidence based practice provides these necessary resources, leading to lower stress, anxiety, and depression. In a similar vein, Bilgin et al. (2025) demonstrated that, despite low positive attitudes toward the work environment, there was a positive but weak correlation between work environment scores and attitudes toward brain drain. This finding indirectly suggests that positive psychosocial resources, such as perceived organizational support (which was low in their study), are so insufficient that they cannot act as an effective buffer against migration intention. In other words, the lack of supportive resources in the work environment undermines the mediating pathway of wellbeing and reinforces the desire to leave both the organization and the country [61]. However, these mediation pathways remain hypothetical in the current evidence base, as most studies relied on self-reported cross-sectional data. Longitudinal studies are required to confirm whether improvements in organizational culture truly lead to sustained increases in workplace happiness over time.

### The detrimental impact of a toxic cultural environment

Conversely, this review starkly illustrates the consequences of a negative organizational culture. A culture tolerating workplace bullying [43, 48] is directly associated with reduced wellbeing, leading to burnout, job dissatisfaction, and absenteeism especially when compounded by perceptions of organizational betrayal. Similarly, cultures characterized by hierarchical rigidity, centralization of power, and individualism [47, 50] devalue employee wellbeing and motivation. The significant negative correlations between value incongruence [49] and wellbeing metrics (burnout, turnover intention) underscore that when an organization’s enacted values conflict with those of its nurses, this may contribute to profound psychological distress ensues. Empirical evidence from the study by Bilgin et al. (2025) clearly demonstrates that a toxic and unsupportive work environment has consequences beyond burnout. In this study, 76.1% of nurses who intended to work abroad had conducted research about the destination country, and 46.9% were proficient in at least one foreign language. These findings indicate that when faced with a toxic organizational culture, nurses not only experience psychological harm but also actively plan to exit the system. Thus, a toxic organizational culture not only diminishes individual wellbeing but also potentially threatens the human capital of the healthcare system [61].

### Cultural specificity and universal levers

While some cultural expressions may vary by context for example, hierarchical structures are often linked to lower wellbeing in Western settings Hwang’s (2019) findings in South Korea suggest that in certain cultural contexts, clear hierarchical roles may provide stability and predictability, which can be perceived positively. This highlights the need for culturally sensitive interpretations of organizational culture, which may reflect cultural norms the underlying universal levers appear to be autonomy, recognition, and a sense of belonging. Tomo & De Simone (2023) identified these as critical for nurses’ wellbeing in Italy, a finding echoed across studies in Egypt, Canada, and the Netherlands. This suggests that while the implementation may be context sensitive, the core human needs for competence, autonomy, and relatedness are suggested as fundamental drivers of nurse wellbeing across healthcare systems globally. An important point is that the study by Bilgin et al. (2025) revealed that the push factors in brain drain are not limited to working conditions alone; living conditions (35.3%) and economic conditions (29.0%) also contribute significantly. This finding confirms that understanding organizational culture and its impact on nurses’ wellbeing must be approached within a broader context of socio-cultural and economic factors. In other words, universal levers such as autonomy and recognition can only be effective when more basic livelihood and welfare needs are also met [61].

### Impact on patient care

The relationship between nurse wellbeing and patient care quality is suggested by the available evidence, though it is important to note that the included studies primarily relied on self-reported measures rather than objective clinical outcomes. Happy and well-supported nurses were more likely to report providing high-quality, patient-centered care, which may translate into improved patient outcomes. For instance, Zhao et al. (2024) found a direct, positive association between organizational learning culture and nurses’ self-reported quality of care. Similarly, research by den Boer et al. (2017) established that patientcare care was a significant predictor of both nurse wellbeing and job satisfaction, creating a potential virtuous cycle where focus on the patient benefits the caregiver and vice versa. Furthermore, a positive work environment, as evidenced by Rondeau & Wagar (2006), leads to higher resident satisfaction in long term care settings. Conversely, organizational factors that diminish wellbeing, such as a prevalent culture of hierarchical rigidity and individualism [47, 50], are detrimental to valuing worker wellbeing and, by extension, patient focused care. It must be emphasized that the evidence underpinning these associations is derived predominantly from self-reported outcomes (e.g., perceived quality of care, resident satisfaction) rather than from objective clinical indicators such as mortality rates, readmission rates, medication errors, or infection rates. The current review did not synthesize robust clinical outcome data, and therefore, causal claims about direct improvements in patient outcomes cannot be made. These findings align with the broader literature confirming a direct link between nurse wellbeing and the quality and safety of healthcare services. This underscores that strategic investment in a positive organizational culture, empowerment, and supportive may be an important component for achieving excellence in patient safety, satisfaction, and clinical outcomes. From a clinical outcomes perspective, the study by Bilgin et al. (2025) provides a serious warning: when attitudes toward brain drain are high (mean score of 56.30), it means that a considerable portion of the skilled workforce is planning to leave the system. This not only disrupts continuity of care but also may directly jeopardizes patient safety. Indeed, brain drain can be regarded as an ultimate and severe consequence of failing to establish a positive organizational culture. Therefore, investing in improving organizational culture may be considered an investment in patient safety and quality of care [61].

### Challenges and barriers

While the cultivation of a positive organizational culture is suggested to be beneficial, the reviewed literature consistently identifies significant systemic barriers that can compromise nurse happiness. These include excessive workloads [52], inadequate managerial support [33, 46], and negative workplace dynamics such as bullying [43, 48] and a punitive climate [47]. These factors collectively contribute to burnout, stress, and diminished job satisfaction, thereby undermining the very foundations of a happy and stable nursing workforce. Addressing these deeply entrenched issues requires a multifaceted and systemic approach. This includes implementing realistic workload management, strengthening leadership and peer support systems, and introducing targeted training programs to foster resilience and effective coping strategies among nursing staff.

The cumulative evidence suggests a need for a decisive shift from fragmented wellness initiatives toward a comprehensive, strategic overhaul of organizational culture. This requires action at multiple levels: healthcare leaders must cultivate competencies in transformational and supportive leadership, actively fostering organizational justice, support, and open communication. At an organizational level, this entails developing a positive and proactive culture by implementing robust antibullying and violence prevention policies, decentralizing decision making to enhance autonomy, and investing in continuous professional development. Furthermore, promising interventions such as establishing regular feedback mechanisms to assess nurse wellbeing, developing leadership and emotional intelligence training for management, encouraging teambuilding activities, and instituting policies that prioritize wellbeing including flexible scheduling and mental health support are critical. Ultimately, for these efforts to be sustained and scalable, policymakers must integrate organizational culture and staff wellbeing as core, incentivized indicators of healthcare quality, placing them on par with patient satisfaction and clinical outcomes.

### Implications of heterogeneity and low-quality evidence

A significant limitation of this review is the high clinical and methodological heterogeneity among the included studies. Variations in the definition of ‘organizational culture’ and the use of diverse instruments (e.g., OPQ, JSS, various leadership scales) to measure both exposure and outcome variables complicate direct comparisons. This heterogeneity, combined with the low quality of the evidence (primarily due to the observational design and potential biases), means that the observed associations may be influenced by unmeasured confounders. Therefore, while the general trend points towards a suggested beneficial effect of positive culture, the specific magnitude of this effect remains uncertain. Future research must employ standardized measures and longitudinal designs to disentangle these complex relationships.

## Conclusion

This systematic review, based on 29 studies predominantly of cross‑sectional design, provides a thematic synthesis suggesting that a positive organizational culture characterized by trust, social support, justice, and transformational leadership is consistently associated with higher nurse wellbeing, lower burnout, and greater retention intentions. However, these conclusions must be interpreted in light of the low to moderate methodological quality of the underlying evidence. Furthermore, while some studies reported associations with self-reported quality of care and resident satisfaction, the review did not synthesize objective clinical outcome data (e.g., mortality, readmission rates, medication errors). The relevance of this review is not to provide definitive causal estimates but rather to: (1) map the existing evidence landscape across diverse global contexts; (2) identify consistent thematic patterns despite methodological heterogeneity; (3) highlight critical gaps in the literature, particularly the lack of longitudinal and experimental studies; and (4) offer evidence‑informed (though low‑certainty) directions for organizational interventions and future research. The predominance of cross-sectional designs means that all reported associations are correlational, not causal. Reverse causality (happy nurses perceiving culture more positively) and unmeasured confounding (e.g., personality, personal life circumstances) cannot be ruled out. Furthermore, substantial heterogeneity in measurement instruments for both culture and outcomes limits the comparability of findings across studies. Response rates in several studies were suboptimal, raising concerns about selection bias. The overall certainty of the available evidence was considered low. Healthcare leaders and policymakers should consider fostering a supportive, ethical, and empowering organizational culture as a potentially promising strategy to enhance nurse wellbeing and retention but as part of a broader workforce strategy, not as a standalone solution. Given the low certainty of evidence, investments in cultural interventions should be accompanied by rigorous local evaluation (e.g., pre-post designs with control groups) to assess their actual impact. Priority areas include: strengthening transformational leadership, ensuring organizational justice, implementing anti-bullying policies, and promoting work life balance.

The most urgent need is for high quality longitudinal cohort studies that track changes in organizational culture and subsequent changes in nurse wellbeing over time, while controlling for individual level confounders. Quasi experimental studies (e.g., interrupted time series or difference in differences) evaluating specific cultural interventions are also needed. Furthermore, the field would benefit from consensus on standardized, validated measurement tools for both organizational culture and nurse happiness to reduce heterogeneity and enable future meta analyses. Future research should also incorporate objective clinical outcome measures to better understand whether improvements in organizational culture translate into measurable improvements in patient safety and clinical quality. In summary, this review suggests that a positive organizational culture is likely beneficial for nurse happiness, but this conclusion rests on low certainty evidence. The findings should inform strategic discussions and hypothesis generation rather than serve as definitive proof of causality. Healthcare leaders should consider cultural interventions as part of a broader retention strategy, but these recommendations rest on low to moderate-certainty evidence. Future longitudinal and experimental research is urgently needed before causal claims or high confidence policy mandates can be made.

### Implications for practice and policy: a quality caveat

Given the low quality of evidence, the following recommendations are offered as directional guidance based on consistent thematic patterns, not as proven causal prescriptions. Healthcare leaders and policymakers may consider fostering a supportive, ethical, and empowering organizational culture as a potentially promising strategy to enhance nurse wellbeing and retention. However, claims that such interventions directly improve objective patient outcomes are not supported by the current evidence base. Such interventions should be: Implemented alongside rigorous local evaluation (e.g., pre-post designs with control groups, or interrupted time series) to assess their actual impact in specific contexts. Treated as part of a broader workforce strategy, not as standalone solutions, given that unmeasured factors (e.g., workload, compensation, staffing ratios) may confound the relationship. Accompanied by commitment to improving the evidence base, including routine collection of longitudinal data and use of standardized, validated measurement tools. Priority areas for action (with low certainty support) include: strengthening transformational leadership, ensuring organizational justice, implementing anti bullying and violence prevention policies, improving perceived organizational support, and promoting work life balance. These are not proven interventions but represent the best available thematic synthesis from low quality evidence.

## Supplementary Information

Below is the link to the electronic supplementary material.

Supplementary Material 1

## Data Availability

No datasets were generated or analysed during the current study.
