# Supplementary material for: The role of organizational culture in nurse wellbeing and work-related psychosocial outcomes: a systematic review
Source: BMC Nurs. 2026 Jul 22;25:991. doi: 10.1186/s12912-026-05086-w (PMC13679156; doi:10.1186/s12912-026-05086-w)
Supplement: Supplementary file 1 [file 12912_2026_5086_MOESM1_ESM.docx]

| Database name | **Search Strategy** |
| --- | --- |
| PubMed | text( ("Organizational Culture"[MeSH Terms] OR "Culture, Organizational"[Title/Abstract] OR "organizational culture"[Title/Abstract] OR "workplace culture"[Title/Abstract] OR "corporate culture"[Title/Abstract] OR "organisational culture"[Title/Abstract])) AND (("Happiness"[MeSH Terms] OR "Job Satisfaction"[MeSH Terms] OR "Burnout, Professional"[MeSH Terms] OR "happiness"[Title/Abstract] OR "well-being"[Title/Abstract] OR "wellbeing"[Title/Abstract]  OR "job satisfaction"[Title/Abstract] OR "work satisfaction"[Title/Abstract] OR "employee satisfaction"[Title/Abstract] OR "nurse satisfaction"[Title/Abstract] OR "psychological well-being"[Title/Abstract] OR "mental well-being"[Title/Abstract] OR "psychological health"[Title/Abstract] OR "burnout"[Title/Abstract] OR "quality of work life"[Title/Abstract]  OR "work engagement"[Title/Abstract] OR "emotional exhaustion"[Title/Abstract])) AND( ("Nurses"[MeSH Terms] OR "Nursing Staff"[MeSH Terms] OR "nurse"[Title/Abstract] OR "nurses"[Title/Abstract] OR "nursing"[Title/Abstract] OR "nursing staff"[Title/Abstract] OR "registered nurse"[Title/Abstract] OR "nurse practitioner"[Title/Abstract] OR "clinical nurse"[Title/Abstract] OR "hospital nurse"[Title/Abstract])) |
| Web of Science | TS=( "organizational culture" OR "workplace culture" OR "corporate culture" OR "organisational culture") ANDTS=( "happiness" OR "well-being" OR "wellbeing" OR "job satisfaction" OR "work satisfaction" OR "employee satisfaction" OR "nurse satisfaction" OR "psychological well-being" OR "mental well-being" OR "psychological health" OR "burnout" OR "quality of work life" OR "work engagement" OR "emotional exhaustion") AND TS=("nurse" OR "nurses" OR "nursing" OR "nursing staff" OR "registered nurse" OR "nurse practitioner" OR "clinical nurse" OR "hospital nurse")  Indexes=SCI-EXPANDED, SSCI, A&HCI, CPCI-S, CPCI-SSH, BKCI-S, BKCI-SSH, ESCI Timespan=All years |
| Scopus | TITLE-ABS-KEY("organizational culture" OR "workplace culture" OR "corporate culture" OR "organisational culture") AND TITLE-ABS-KEY("happiness" OR "well-being" OR "wellbeing" OR "job satisfaction" OR "work satisfaction" OR "employee satisfaction" OR "nurse satisfaction" OR "psychological well-being" OR "mental well-being" OR "psychological health" OR "burnout" OR "quality of work life" OR "work engagement" OR "emotional exhaustion") AND TITLE-ABS-KEY("nurse" OR "nurses" OR "nursing" OR "nursing staff" OR "registered nurse" OR "nurse practitioner" OR "clinical nurse" OR "hospital nurse") |

Database 1: PubMed

Search Date: January 2026

Language Restriction: English

Database 2: Web of Science

Search Date: January 2026

Language Restriction: English

Database 3: Scopus

Search Date: January 2026

Language Restriction: English
